# Bile acid-induced modulation of virus replication

**DOI:** 10.1186/2047-783X-19-S1-S27

**Published:** 2014-06-19

**Authors:** Anna-Kathrin Schupp, Dirk Graf

**Affiliations:** 1 Clinic of Gastroenterology, Hepatology and Infectious Diseases, Heinrich Heine University, 40225 Düsseldorf, Germany

## 

Bile acids function as signaling molecules. As such they are involved in cellular immunological processes mainly mediating anti-inflammatory effects. They suppress cell-mediated innate and adaptive immunity through different signaling pathways, e.g. mediated by the nuclear bile acid receptor farnesoid x receptor (FXR) or the G-coupled receptor TGR5 at the outer plasma membrane. In the past it became increasingly evident that bile acids also modulate viral replication, which is reviewed herein.

## Bile acids and interferon signaling

Interferons are central antiviral cytokines, which in part transmit their antiviral capacity via expression of antiviral proteins like ds-RNA-activated protein kinase (PKR), 2´5´oligoadenylate synthase (OAS) and myxovirus resistance protein A (MxA). Bile acids impair interferon-α and β signaling in hepatocytes, NK cells, macrophages and lymphocytes. Results of our group showed that in case of hepatocytes hydrophobic bile acids blunt IFNα-dependent JAK1 and Tyk2 activation. These effects of bile acids on IFN-signaling may limit the therapeutic use of IFNs in hepatitis B and hepatitis C treatment [1].

## Bile acids and HCV

The success of HCV therapy with IFN is dependent on serum bile acid levels, as in patients with high serum levels IFN therapy shows higher failing rates. Chang et al. showed that low concentrations of unconjugated bile acids [deoxycholic acid (DCA)/ chenodeoxycholic acid (CDC)] and very high concentrations of the conjugated bile acid glycochenodeoxycholic acid (GCDC) increase HCV RNA replication in HCV genotype 1b replicon-harboring GS4.1 cells. Importantly, high concentrations of the therapeutically used hydrophilic ursodeoxycholic acid may also induce a pro-viral effect [2], however different studies in HCV patients show lower transaminases levels without changes in virus load in response to ursodeoxycholic acid treatment. In another study, the proviral effect of DCA and CDC on HCV was shown in HCV genotype 1b transfected Huh-7 cells, where conjugated forms of these bile acids do not mediate pro-viral effects [3]. A disadvantage of both studies is the usage of cells that did not express the bile acid transporter sodium taurocholate cotransporting polypeptide (NTCP), which is essential for import of conjugated bile acids, so that the effect of conjugated bile acids on HCV replication could not be analyzed clearly. Both studies showed that the pro-viral effect of bile acids is dependent on FXR activation. Furthermore inhibition of FXR activity reduced HCV RNA replication of genotype R1a and R1b but not R2a independently of bile acid treatment [3]. In HCV infected cells extracellular signal- regulated kinase (Erk) is activated and lead to cell cycles with extended S-phases, which had a positive effect on HCV replication. Patton et al. showed that the pro-viral effect of bile acids on HCV genotype 1a and 1b is dependent on epidermal growth factor receptor (EGFR)/ERK pathway activation that results in a prolonged S phase [4]. Only one study characterized the pro-viral effect of bile acids in HCV life cycle and showed an induction of viral RNA-replication, without effecting viral entry or RNA translation. Furthermore, bile acids did not increase the abundance of cell free virus particles, but particles seem to be more infective than virus particles from untreated cells [5]. The choice of the viral system, the used cell lines or the usage of partial or whole HCV genome seem to have a significant influence on bile acid effects on virus replication, as genotype 2a replicon harboring cells show no pro-viral effect in response to bile acids [3], while HCV replication could be induced by bile acids after HCV genotype 2a genome transfection [5].

## Bile acids and HBV

Hepatitis B virus (HBV) only replicates in the liver, probably due to the fact that NTCP is the entry receptor for HBV and replication is dependent on liver enriched transcription factors as hepatocyte nuclear factor 4α, liver receptor homolog 1, estrogen-related receptor or heterodimers of retinoid X receptor (RXRα) with FXRα or peroxisome proliferator- activated receptor [6]. Two binding sites of FXR and RXRα heterodimers are localized in the HBV enhancer 2 and core promoter region. Transfection of Huh-7 cells with FXRα and a luciferase construct carrying the enhancer 2 and core promoter region of HBV lead to an induction of luciferase activity, which can be increased by CDC and RXRα co-transfection [7]. Additional to FXR dependent activation of HBV enhancer 2 by bile acids, CDC treatment leads to JNK/cJun and HBV enhancer 1 activation that also result in HBV enhancer 2 induction. In contrast, bile acids induce small heterodimer partner (SHP), which mediates inhibitory effects on HBV replication. Recently it was shown that the activation of RXRα or FXR alone is sufficient to induce replication of HBV [8]. An *in vivo* study with HBV transgenic mice, which were fed with 1% cholic acid, showed only slight effects of bile acid feeding on HBV transcripts. Notably, bile acids feeding only induced HBV in male mice, while it had no effect on virus replication in female mice [6].

In summary, bile acids activate multiple signaling pathways and transcription factors to induce or inhibit virus replication in the liver and the intestine. It would be interesting to determine if other viruses that preferentially replicate in the liver, such as hepatitis A virus, hepatitis D virus and hepatitis E virus, are also affected by bile acids and have developed mechanisms to survive or even benefit from bile acid signaling. Moreover, bile acids modulate cell signaling in the gut and immune cells, so that further effects of bile acids on virus replication are also probable.
